# Supplementary material for: Genome-Wide Mining of the Tandem Duplicated Type III Polyketide Synthases and Their Expression, Structure Analysis of Senna tora
Source: Int J Mol Sci. 2023 Mar 2;24(5):4837. doi: 10.3390/ijms24054837 (PMC10003783; doi:10.3390/ijms24054837)
Supplement: Supplementary file 1 [file ijms-24-04837-s001.zip › Supplementary figures.pdf]

CHS-L2 **Ramachandran Plot**

saves

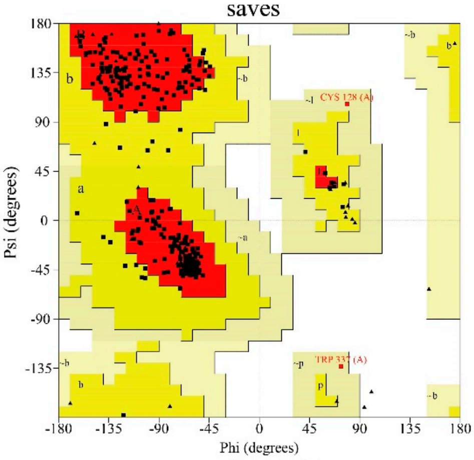

Plot statistics

Residues in most favoured regions [A,B,L]	303	92.9%
Residues in additional allowed regions [a,b,l,p]	21	6.4%
Residues in generously allowed regions [~a,~b,~l,~p]	2	0.6%
Residues in disallowed regions	0	0.0%
Number of non-glycine and non-proline residues	326	100.0%
Number of end-residues (excl. Gly and Pro)	2	
Number of glycine residues (shown as triangles)	36	
Number of proline residues	21	
Total number of residues	385	

Based on an analysis of 118 structures of resolution of at least 2.0 Angstroms and R-factor no greater than 20%, a good quality model would be expected to have over 90% in the most favoured regions.

CHS-L3 **Ramachandran Plot**

saves

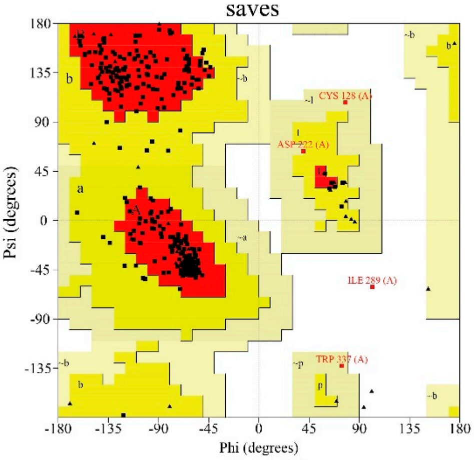

Plot statistics

Residues in most favoured regions [A,B,L]	303	92.4%
Residues in additional allowed regions [a,b,l,p]	21	6.4%
Residues in generously allowed regions [~a,~b,~l,~p]	3	0.9%
Residues in disallowed regions	1	0.3%
Number of non-glycine and non-proline residues	328	100.0%
Number of end-residues (excl. Gly and Pro)	2	
Number of glycine residues (shown as triangles)	34	
Number of proline residues	21	
Total number of residues	385	

Based on an analysis of 118 structures of resolution of at least 2.0 Angstroms and R-factor no greater than 20%, a good quality model would be expected to have over 90% in the most favoured regions.

CHS-L5 **Ramachandran Plot**

saves

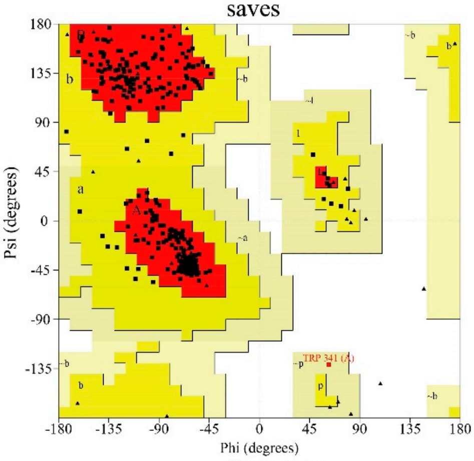

Plot statistics

Residues in most favoured regions [A,B,L]	317	94.1%
Residues in additional allowed regions [a,b,l,p]	19	5.6%
Residues in generously allowed regions [~a,~b,~l,~p]	1	0.3%
Residues in disallowed regions	0	0.0%
Number of non-glycine and non-proline residues	337	100.0%
Number of end-residues (excl. Gly and Pro)	2	
Number of glycine residues (shown as triangles)	33	
Number of proline residues	19	
Total number of residues	391	

Based on an analysis of 118 structures of resolution of at least 2.0 Angstroms and R-factor no greater than 20%, a good quality model would be expected to have over 90% in the most favoured regions.

CHS-L6 **Ramachandran Plot**

saves

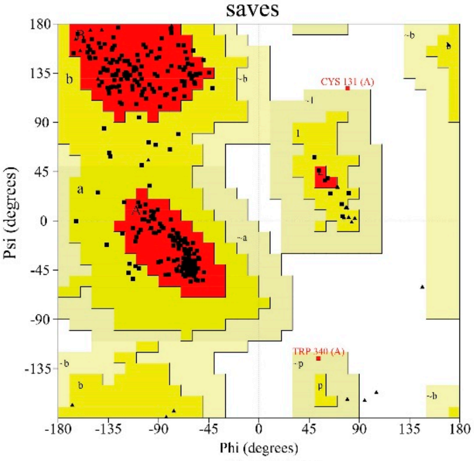

Plot statistics

Residues in most favoured regions [A,B,L]	312	92.3%
Residues in additional allowed regions [a,b,l,p]	24	7.1%
Residues in generously allowed regions [~a,~b,~l,~p]	1	0.3%
Residues in disallowed regions	1	0.3%
Number of non-glycine and non-proline residues	338	100.0%
Number of end-residues (excl. Gly and Pro)	2	
Number of glycine residues (shown as triangles)	31	
Number of proline residues	19	
Total number of residues	390	

Based on an analysis of 118 structures of resolution of at least 2.0 Angstroms and R-factor no greater than 20%, a good quality model would be expected to have over 90% in the most favoured regions.

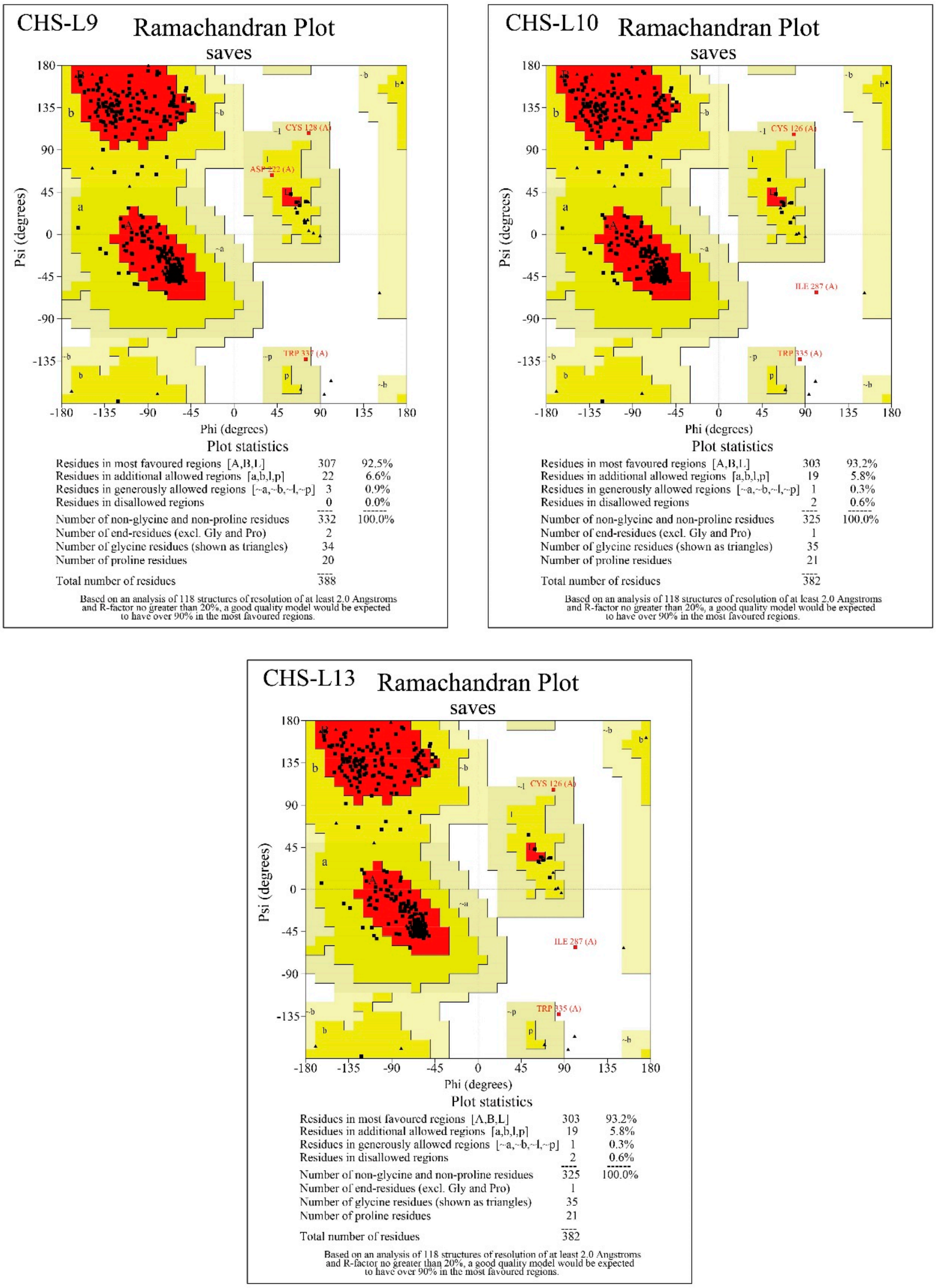

Figure S1. Ramachandran plots of the seven predicted CHS-L protein models in *Senna tora*.

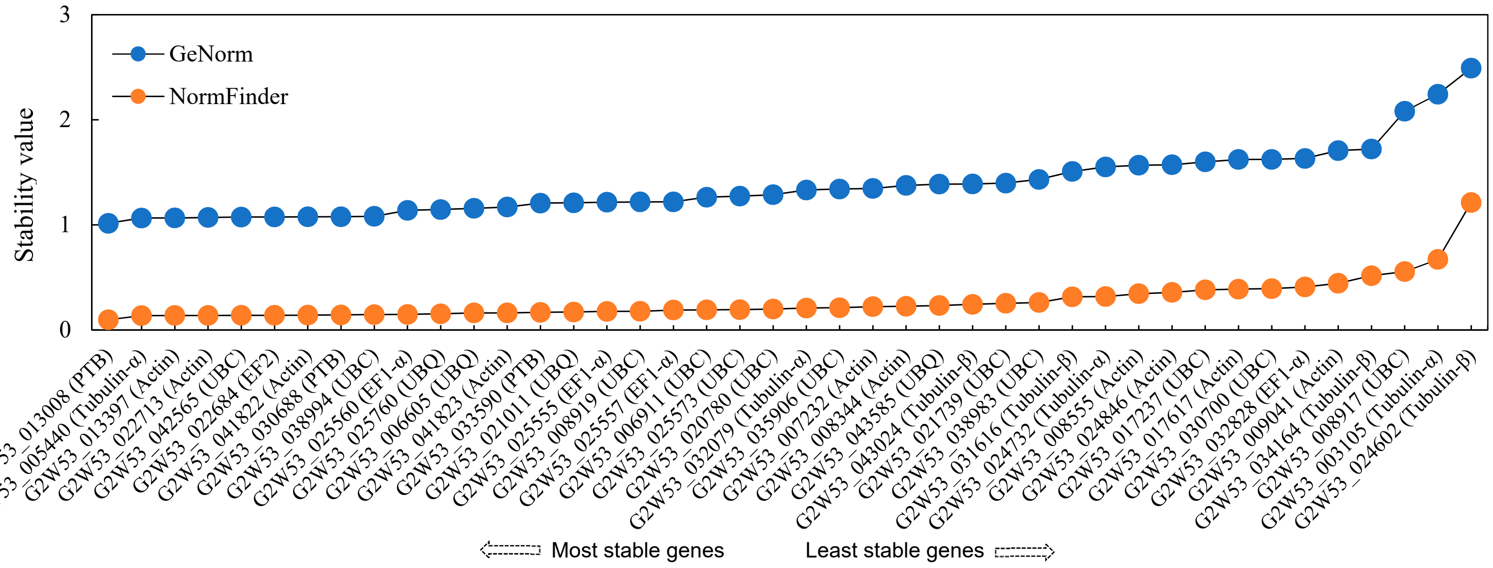

Figure S2. Expression stability values of 42 commonly used reference genes at transcriptome levels in *Senna tora*. We used GeNorm (version 3.4) and NormFinder (version 0.953) software to test the expression stability of genes, depending on Log2 transformed expression levels (TPM values). The lower stability value is, the higher stability the reference gene has. Results showed that the G2W53_013008 (PTB) gene was the most stable one. PTB: Polypyrimidine tract-binding protein-like protein; UBC: Ubiquitin-conjugating enzyme; UBQ: Polyubiquitin; EF2: Elongation factor 2; EF1-α: Elongation factor 1-alpha; TPM: Transcripts per million.
